# Invasive species modulate the structure and stability of a multilayer mutualistic network

**DOI:** 10.1098/rspb.2023.0132

**Published:** 2023-06-28

**Authors:** Agustin Vitali, Sofía Ruiz-Suarez, Diego P. Vázquez, Matthias Schleuning, Mariano A. Rodríguez-Cabal, Yamila Sasal, Shai Pilosof

**Affiliations:** ^1^ Department of Life Sciences, Ben-Gurion University of the Negev, Beer-Sheva, Israel; ^2^ Grupo de Ecología de Invasiones, INIBIOMA, Universidad Nacional del Comahue, CONICET. San Carlos de Bariloche, Río Negro, Argentina; ^3^ Instituto Argentino de Investigaciones de las Zonas Áridas, CONICET - Universidad Nacional de Cuyo, Mendoza, Argentina; ^4^ Facultad de Ciencias Exactas y Naturales, Universidad Nacional de Cuyo, Mendoza, Argentina; ^5^ Senckenberg Biodiversity and Climate Research Centre (SBiK-F), Frankfurt am Main, Germany; ^6^ Rubenstein School of Environment and Natural Resources, University of Vermont, Burlington, Vermont 05405, USA; ^7^ The Goldman Sonnenfeldt School of Sustainability and Climate Change, Ben-Gurion University of the Negev, Be’er Sheva, Israel

**Keywords:** ecological networks, multilayer networks, mutualistic interactions, community stability, invasive species

## Abstract

Species interactions are critical for maintaining community structure and dynamics, but the effects of invasive species on multitrophic networks remain poorly understood. We leveraged an ongoing invasion scenario in Patagonia, Argentina, to explore how non-native ungulates affect multitrophic networks. Ungulates disrupt a hummingbird–mistletoe–marsupial keystone interaction, which alters community composition. We sampled pollination and seed dispersal interactions in intact and invaded sites. We constructed pollination and seed dispersal networks for each site, which we connected via shared plants. We calculated pollination-seed dispersal connectivity, identified clusters of highly connected species, and quantified species’ roles in connecting species clusters. To link structural variation to stability, we quantified network tolerance to single random species removal (disturbance propagation) and sequential species removal (robustness) using a stochastic coextinction model. Ungulates reduced the connectivity between pollination and seed dispersal and produced fewer clusters with a skewed size distribution. Moreover, species shifted their structural role, fragmenting the network by reducing the ‘bridges’ among species clusters. These structural changes altered the dynamics of cascading effects, increasing disturbance propagation and reducing network robustness. Our results highlight invasive species’ role in altering community structure and subsequent stability in multitrophic communities.

## 1. Introduction

Species interactions are the backbone of the structure and dynamics of communities [1,2]. Therefore, it is crucial to comprehend how disruptions in species interactions impact community stability, particularly in the face of anthropogenic effects. While a considerable amount of research has been conducted on the link between structure and stability in networks representing one type of ecological interaction (i.e. monotrophic networks, such as food webs) [3,4], species in natural communities typically engage in multitrophic interactions. For example, flowering plants rely on both pollination and seed dispersal. However, few studies have explored the relationship between structure and stability in multitrophic communities, and those primarily used theoretical modelling and simulated data [5–8]. Moreover, it is uncertain how the relationship between structure and stability operates in disturbed habitats, such as those invaded by non-native species. Therefore, further research is needed to directly compare the structure-stability relationship in disturbed and undisturbed natural sites, particularly given the current rates of species invasion and biodiversity loss [9]. An open question, therefore, remains regarding how invasive species impact the response of multitrophic communities to perturbations in nature.

The link between the structure and stability of ecological communities is well studied in monotrophic networks [10,11]. For instance, a modular structure—in which species within modules interact more frequently with each other than with species from other modules—increases stability because perturbations tend to be locked within modules before spreading to others [12]. To date, however, modularity has rarely been studied in multitrophic networks. Moreover, species play different structural roles according to the distribution of their interactions across partners within and among modules, influencing network cohesion [13] and the dynamics of cascading effects [14,15]. For example, connector species that link modules may promote disturbance propagation across them, although the same species can play different structural roles when involved in different trophic interactions. Moreover, when multiple types of interactions are considered, the connectivity between different trophic groups may also affect the propagation of disturbances. For instance, the robustness of parrot communities was found to increase when antagonistic and mutualistic interactions with plants were considered [7].

**Figure 1.**
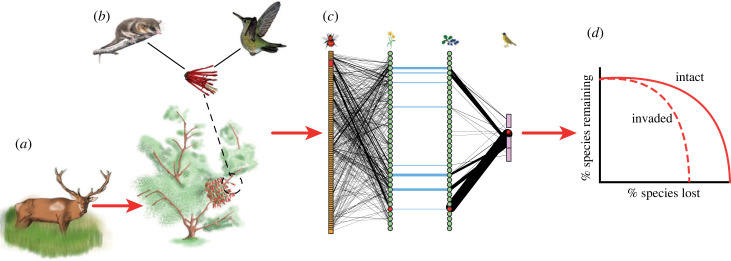
Study system and hypothesis. The mistletoe (*Tristerix corymbosus*) grows on its host *Aristotelia chilensis* and provides nectar to the migratory hummingbird (*Sephanoides sephaniodes*) and fruits for the seed disperser marsupial (*Dromiciops gliroides*), sustaining their populations. The hummingbird pollinates almost 20% of woody plants in summer [18,19] and the marsupial disperses up to 58% of fleshy fruited species, providing a crucial service for plants with large fruits that native birds do not disperse [20,21]. In addition, the marsupial is the only seed disperser of the mistletoe, facilitating the seeds’ placement on branches of an appropriate host [20]. Because most of the plants depend on a few mutualistic partners to reproduce and disperse in the Patagonian forest [18], the reduction in the abundance or loss of generalist mutualistic species, such as the hummingbird and seed disperser marsupial, could trigger extinction events in the community [22]. (*a*). Disruption by non-native ungulates leads to the extinction of the hummingbird–mistletoe–marsupial keystone interaction by consuming the primary host of the mistletoe, and via local extinction of the marsupial [23] (*b*). The disruption of this keystone interaction produces cascading effects that reduce the number of species and interactions in pollination and seed dispersal networks and alter the richness and abundance of foliage arthropods [24,25]. However, the relationship between the multitrophic structure and stability remains unknown. We hypothesized that non-native ungulates would alter the structure of the pollinator–plant–seed disperser multilayer network (*c*), affecting the stability of the community (*d*). The area under the curve in (*d*) is used as a measure for robustness (e.g. figure 4*b*). The solid and dashed lines are the robustness curves (toy example) of the intact and invaded networks. The area under the dashed curve is smaller, indicating a reduction in robustness following invasion. Complete description of the network in (*c*) is in figure S4 in the electronic supplementary material. Panels (*a*) and (*b*) were adapted from [23].

Early works emphasized the importance of keystone species to the stability of ecological communities. Analogously to keystone species, keystone interactions are those that determine structural and functional properties of communities [16,17]. For instance, in the temperate forest of Patagonia, a multitrophic keystone interaction between a hummingbird, a mistletoe and a seed disperser marsupial was identified (figure 1) [23,24]. The mistletoe provides a unique nectar resource for the generalist hummingbird during winter, supporting resident populations in the forest [18,26]. Its fruits allow the marsupial to increase its abundance and sustain the dispersal of other fleshy fruited species [27,28]. Non-native ungulates (deer and cattle) disrupt this keystone interaction via herbivory on the main host of the mistletoe, *Aristotelia chilensis*, and by causing changes to the vegetation structure. This disruption reduces the complexity of pollination and seed dispersal networks [24].

While it is necessary to consider keystone interactions when studying the link between structure and stability [16,29], their disruption also needs to be better understood, particularly for multitrophic networks. To address this gap, we leveraged the ongoing ungulate invasion disrupting the Patagonian hummingbird-mistletoe-marsupial keystone interaction (figure 1). An ideal framework to link multitrophic interaction networks is multilayer networks [30]. While ecological multilayer networks are increasingly used, how their structure relates to stability is still grossly understudied.

This work aims to study how the subsequent loss of a keystone interaction due to invasive species affects the structure and stability of multitrophic networks. We address this goal using multilayer network analysis [30]. We hypothesized that non-native ungulates would alter the structure of the network. Previous studies found that non-native ungulates disrupt mutualistic interactions [31] and reduce the complexity of pollination and seed dispersal networks [24]. In our networks, the invaded network contained fewer species than the intact (24 versus 37 plants, 67 versus 95 pollinators, 4 versus 4 seed dispersers; electronic supplementary material, table S1). Therefore, we expected higher connectivity between the trophic levels and a higher number of modules in the intact than in the invaded network. In addition, we expected species to change their structural role between the intact and invaded networks, as has been recorded for other types of disturbance [32]. We tested this hypothesis by measuring connectivity between trophic levels, modularity, and the structural role of species. Second, we hypothesized that by disrupting the network structure, non-native ungulates would alter stability. That is, the dynamics of cascading effects following a perturbation. We expected a lower tolerance to removing one randomly selected species (i.e. higher disturbance propagation) and to the sequential removal of species (i.e. lower network robustness) in the invaded than in the intact network.

## 2. Material and methods

### (a) Study area

The study was conducted in the Llao Llao Municipal Reserve ($41^{\circ }9^{\prime }$ S, $71^{\circ }18^{\prime }$ W) and Nahuel Huapi National Park in Patagonia, Argentina ($40^{\circ }58^{\prime }$ S, $71^{\circ }31^{\prime }$ W), located at the Subantarctic biogeographical region [33]. Currently, 56% of the area of the Nahuel Huapi National Park is occupied by the non-native ungulates red deer (*Cervus elaphus*), dama deer (*Dama dama*) and domestic cattle (*Bos taurus*), which are the most abundant ungulates in the forest [34].

We leveraged this ongoing invasion scenario to compare non-invaded and invaded sites. We selected four 1 ha sites representing native plant communities, separated by more than 2 km (‘Site description and sampling effort’ in the electronic supplementary material). The two ‘intact’ sites had the keystone interaction and no records of herbivory from ungulates. The two ‘invaded’ sites were characterized by herbivory by non-native ungulates for over 100 years, and had historical records of the keystone interaction [35,36]. Invaded sites have previous and current records of adult mistletoes and previous records of *D. gliroides*. The keystone interaction is ecologically extinct at invaded sites due to (i) the absence of mistletoe recruitment triggered by the herbivory of its main host (*A. chilensis*) and (ii) changes to the vegetation, which produced the local extinction of its only seed disperser, *D. gliroides* [23] (tables S2–S3 in the electronic supplementary material).

### (b) Data collection

We used data from Vitali *et al.* [24]. Briefly, pollinator–plant and frugivore-plant interactions were recorded for all flowering and fleshy-fruited plant species during two flowering and fruiting seasons (2017–2018 and 2018–2019) at each site. Pollinator–plant interactions were recorded by conducting 10 min censuses per plant, with interactions being considered when the reproductive structure of the flower was touched by the visitor. Seed dispersal by birds was recorded by conducting 1 h censuses per plant, with interactions being considered when the bird swallowed the fruit. Seed dispersal by the marsupial *D. gliroides* was recorded by capturing individuals using Tomahawk traps, collecting faeces, and identifying the consumed seeds. In addition, infrared camera traps (Bushnell trophy cam) were used to improve sampling completeness of interactions by accumulating more observation hours per plant species. Sampling effects were controlled by standardizing the sampling effort per species. The same number of censuses of each plant species was conducted among sites (when the same species were present) to ensure consistent sampling effort. Similarly, the sampling effort for the marsupial seed disperser and the use of camera traps among sites was standardized by trapping for the same number of days and filming each plant species for the same amount of time (electronic supplementary material, tables S5 and S6). The accumulation curves of species interaction richness indicated high and consistent sampling completeness (electronic supplementary material, figures S2 and S3). Details on the sampling effort can be found in ‘Sampling effort’ in the electronic supplementary material.

### (c) Network construction

Our goal was to compare intact and invaded sites. Therefore, we pooled interaction data across sites and seasons within each site type to increase our confidence in capturing the maximum number of interactions. We built ‘invaded’ and ‘intact’ multilayer networks. Each network included four components as follows (electronic supplementary material, figure S4) [30,37].

1. Two layers representing pollination (*α*) and seed dispersal (*β*) interactions.
2. Three sets of nodes representing pollinator (in layer *α*), seed disperser (in layer *β*) and plant (both layers) species.
3. Intralayer weighted links connecting plant *j* with pollinator *i*, defined as $w_{\;ji}^{\alpha }=f_{\;ji}/V^{\alpha }$, where *f*_*ji*_ denote the number of visits of *i* to *j* and $V^{\alpha }$ denotes the total number of visits in layer *α*. Similarly, intralayer weighted links connecting plant *j* with seed disperser *k*, analogously defined as $w_{\;jk}^{\alpha }=f_{\;jk}/V^{\beta }$.
4. Interlayer weighted links connecting each plant species with itself when it was both pollinated and dispersed by animals. Biologically, interlayer links encode the extent to which plants mediate the biomass or energy flow between pollination and seed dispersal. We therefore defined an indirect link as a walk from a node in one layer to a node in another via a plant (electronic supplementary material, figure S5). We then calculated interlayer link weight as $w_{\;j}^{\alpha \beta }=n_{\;j}/L_{\mathrm{tot}}$, where *n*_*j*_ is the total number of indirect links between any pollinator and seed disperser species mediated by the plant species *j*, and *L*_tot_ is the total number of indirect links connecting all pollinator and seed disperser species.

Note that the weight of both intralayer and interlayer links ranged between 0 and 1. This places the two link types on the same scale, ensuring that the calculation of network properties (e.g., modularity) is not *a priori* biased towards any of these [30,38].

### (d) Data analysis

#### (i) Connectivity between pollination and seed dispersal

We conducted two analyses to estimate the connectivity between pollination and seed dispersal. First, we recorded the presence of plant species *j* connecting both mutualisms and estimated its interlayer connectivity. We used two generalized linear mixed models (GLMMs) to test whether plant species’ presence and interlayer connectivity differed between the intact and invaded network. Our response variables were the presence/absence of a plant connecting layers and its interlayer link weight ($w_{\;j}^{\alpha \beta }$). The explanatory variable was network types (intact versus invaded). We included ‘species’ as a random factor to control for species-specific differences. We used binomial and gamma distributions with an inverse link function because the response variables were either binary (binomial) or had positive continuous values that are not normally distributed (gamma) [39]. We performed the analyses with the lme4 and glmmTMB packages in R [40–42]. Second, we assessed if non-native ungulates change the connection between pollinator and seed disperser species. To do this, we built a binary matrix for the intact and invaded networks, encoding the existence of at least one indirect link between both trophic groups and calculated the proportion of indirect links per pollinator and seed disperser species (electronic supplementary material, figure S6). We used a binary matrix because only a few pollinator and seed disperser species shared more than one plant species. Then, we performed a GLMM for each trophic group with the proportion of indirect links per species as a response variable and network types as an explanatory variable. We used ‘species’ as a random factor and gamma distribution with an inverse link function distribution for the response variable.

#### (ii) Network modularity and structural species role

To evaluate network fragmentation, we identified the number of modules and the role of species in assembling the network structure. First, for each type of network, we identified groups of tightly connected species with a modularity analysis using Infomap [43]. Infomap detects an optimal network partition based on the movement of a random walker on the network and is specifically designed for multilayer networks [43,44] (see ‘Modularity’ in the electronic supplementary material). To assess if the observed network structure is a result of random processes we compared the observed number of modules to values generated by shuffling each network with a null model. To shuffle the networks, we used the ‘r2dtable’ algorithm (vegan package within R [45]), which we modified to account for the multilayer network structure. The algorithm shuffles the individual interactions within each layer, while preserving the total number of interactions per species (row and column marginal sums). Then, we calculated a *p*-value based on the proportion of the shuffled values that are larger or smaller than the observed value using a two-tailed *t*-test. Significant results indicate that the observed structure of each type of network is not random.

The spread of a perturbation between modules depends on their pattern of connectivity. We used the modularity results to assess species’ role in network connectivity and how invasion changed their role. To do this, we assigned species roles by calculating their ‘position’ with respect to other species in their module (within–module degree, ‘*z*’) and in other modules (between-module connectivity, *c*) [13] (see ‘Structural role of species’ in the electronic supplementary material and figure S8). Peripheral species are primarily linked within their module (*z* ≤ 2.5 and *c* ≤ 0.62). Connector species are primarily linked to species in other modules (*z* ≤ 2.5 and *c* > 0.62). Module hub species are more connected than peripherals but still primarily within their module (*z* > 2.5 and *c* ≤ 0.62). Network hub species are more connected than connectors and are primarily linked to species in other modules (*z* > 2.5 and *c* > 0.62). Because modularity assigns nodes in layers to modules, plants that appear in more than one layer can be assigned to more than one module (a plant *j* in layer *α* can be assigned to a different module than the same plant *j* in layer *β*). Therefore, each plant that occurs in both layers was assigned two structural roles (which could be the same or not). Ecologically, this also makes sense because the same plant can have different ecological roles in different layers; for example, be highly connected in the pollination layer but peripheral in the dispersal layer. We recorded changes in the structural role of species that occurred in the intact and invaded networks. For example, a pollinator could be a connector in the intact network but peripheral in the invaded network.

#### (iii) Network stability

To capture different stability aspects in the intact and invaded networks, we compared their tolerance to single and sequential species removal [46–48]. The former allows us to estimate the propagation after a particular disturbance event (e.g. extinction of a species), while the latter estimates the tolerance of the network to continued disturbances before collapsing (also called robustness analysis [46]). To simulate both extinction scenarios, we used a stochastic coextinction model [47], which we modified to include three different trophic groups. This method is optimal for evaluating extinction cascades because it considers the intrinsic demographic dependence of each species on mutualism and incorporates the mutual dependence of each species on its mutualistic partners [47] (see ‘Additional explanations of data analysis’ in the electronic supplementary material). To link modularity and species roles to stability, we removed species according to species roles rather than by degree as is commonly done.

In each simulation of a single species removal, we considered that the community reached an equilibrium when coextinctions did not propagate any longer after removing the target species [47]. Once equilibrium was reached, we calculated the total percentage of extinct species (*E*). Higher values of *E* indicate higher disturbance propagation. As the model is stochastic, we repeated the removal of each species for each network 100 times and used the average value of *E*. We used a GLMM to test whether *E* was affected by the intact and invaded network, the structural role of the removed species (peripheral, connector, module hub and network hub), or the interaction between them. We included ‘Species’ as a random factor and assumed a gamma distribution in the model. In addition, to compare among different levels of the explanatory variables, we performed a multiple comparison test. Analyses were performed using the lme4, multcomp and lsmeans packages in R [49]. See ‘Stochastic coextinction models’ in the electronic supplementary material, for more details.

In the robustness analysis, we removed species sequentially under three scenarios, according to their structural role, which is an estimate of the species’ ability to maintain network cohesiveness: (i) removal from the most to the least connected structural role (order: network hub, module hub, connector, peripheral); (ii) the opposite order; and (iii) random removal order. In scenarios (i) and (ii), the order of removal within each role was random. We used the stochastic extinction model explained above, but this time, we removed the first species in each scenario. Once the community reached equilibrium, we removed the next species, and so on, until all species of the network became extinct. We calculated the robustness of the network to species extinction as the area under the curve (AUC) of the proportion of species remaining in the network (*y*-axis) as a function of the proportion of species removed (*x*-axis). Higher values of AUC suggest higher tolerance to sequential loss of species [50]. For each network and scenario combination, we ran 1000 simulations and calculated the AUC. Then, we used a GLM model to test if AUC (response variable assuming a gamma distribution) was affected by the intact and invaded network, removal scenario, and their interaction. In addition, we performed a multiple comparison test to compare different levels of the explanatory variables. Analyses were performed using the lme4 and lsmeans packages in R. Finally, we controlled for network size because it is correlated with other structural properties [51] and could therefore affect *E* and AUC. We did that by bootstrapping the largest network. Network size affected *E* but not AUC (see ‘Extinction analysis controlling for network size’ in the electronic supplementary material).

## 3. Results

### (a) Invasive ungulates altered network connectivity

Non-native ungulates reduced the connectivity between pollination and seed dispersal. The presence of plant species connecting pollination and seed dispersal mutualism was almost double in the intact (eight species) than in the invaded (five species) network (GLMM, *z* = 2.617, *p* < 0.01). Nevertheless, they represented almost the same proportion of species (22% and 21% of total species, respectively). In addition, we did not find differences in plants’ interlayer link weights between the intact and invaded network (GLMM, *z* = 0.358, *p* = 0.721), suggesting that the ability of plants to connect between trophic levels is similar regardless of the presence of non-native ungulates. Conversely, the proportion of indirect links per pollinator (GLMM, *z* = 5.642, *p* < 0.001) and seed disperser species (GLMM, *z* = 0.79, *p* = 0.42) were greater in the intact network, indicating a stronger connection between both trophic groups. In the intact network, pollinators and seed dispersers had 56% and 59% more indirect links (pol = 2.75 ± 0.14; disp = 34.25 ± 6.8) than in the invaded network (pol = 1.76 ± 0.17; disp = 21.6 ± 6.7). Moreover, the identity of indirect links between pollinators and seed dispersers was highly dissimilar between network types (Jaccard dissimilarity index = 0.82), indicating that the differences between these networks were not solely numeric.

### (b) Invasive ungulates reduced module number and altered the structural role of species

**Figure 2.**
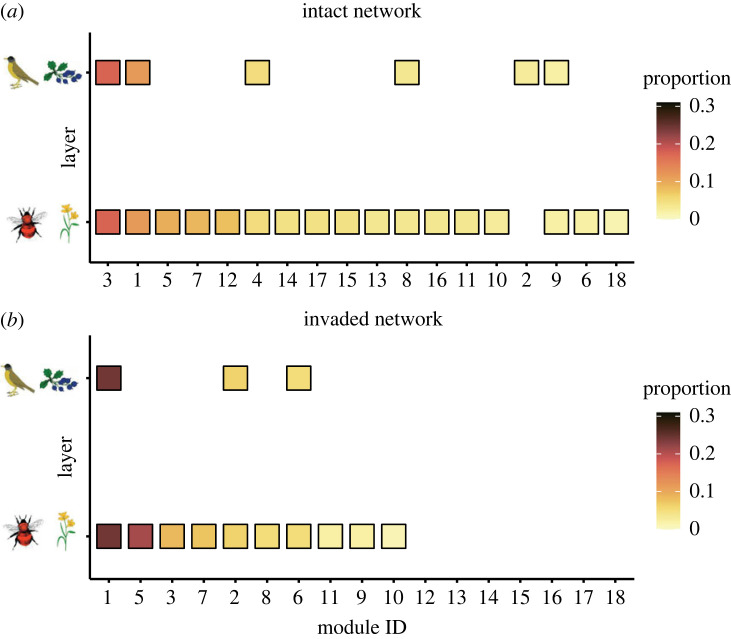
Modular structure differs between the networks. Module-layer combinations are shown for the (*a*) intact and (*b*) invaded networks. Modules can span layers, and each square represents the occurrence of a specific module in a layer. While most modules were limited to a single layer, a few spanned both layers (e.g. module 1 in both networks). The squares’ colour depicts the proportion of species in the network assigned to the module. Modules are ordered from higher to lower numbers of species (from left to right). Module IDs are assigned randomly and independently for the two networks (e.g. module 1 in the intact network is not the same as in the invaded).

The observed number of modules differed from that of the shuffled networks in the intact and invaded networks (*P* < 0.001; electronic supplementary material, figure S7), indicating that their modular structure was non-random. In both networks, five (28%) and three (33%) modules contained species from the three species groups, respectively. However, the intact network had 80% more modules than the invaded network (18 versus 10) (figure 2).

Most species, mainly pollinators, were classified as peripherals in both networks, but the percentage of peripheral species was lower in the intact than in the invaded network (57% and 71%, respectively; electronic supplementary material, figure S9). In contrast, the percentage of connector species was higher in the intact (41%) than in the invaded network (27%), which indicates less connection among modules and, therefore, a more fragmented network in the presence of non-native ungulates. Specifically, two seed disperser species acted as connectors (*D. gliroides* and the generalist bird *Elaenia albiceps*) in the intact network. However, no seed dispersers were connectors in the invaded network. Only plants acted as module hubs in both the intact and invaded networks. However, we detected network hubs only in the intact network: the plants *Alstromeria aurea* and *Schinus patagonicus*.

**Figure 3.**
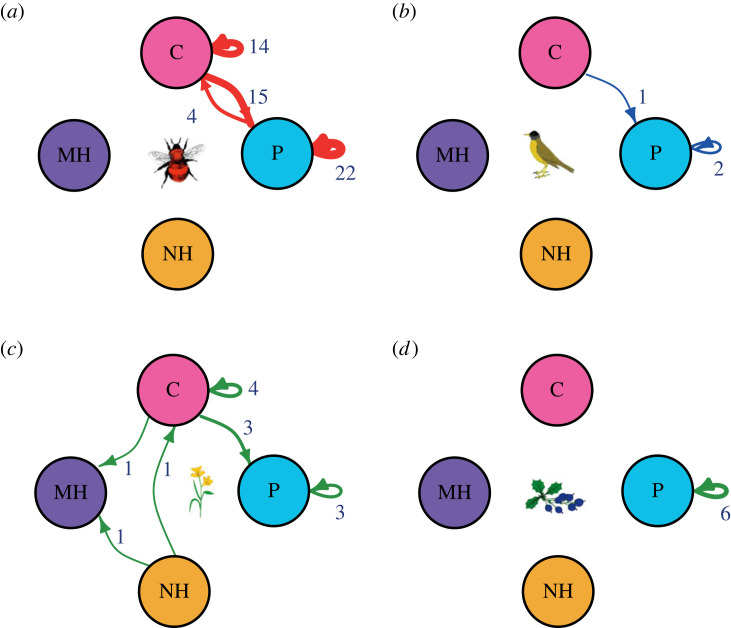
Changes in species structural roles. Circles indicate species’ structural roles: P = peripheral (light blue), C = connector (pink), MH = module hub (purple), NH = network hub (orange). Arrows represent changes in the structural role. The origin and end of arrows represent the role of species in the intact (from) and the invaded (to) networks, respectively. Self-loops indicate no change in the structural role. Values in each arrow indicate the number of species that experienced (or not, in the case of self-loops) the change in their structural role. Panels represent different trophic groups: (*a*) pollinators, (*b*) seed dispersers, (*c*) plants in the pollination layer and (*d*) plants in the seed dispersal layer. Only species shared between network types are included in the calculations and figure.

Ungulate invasion changed the structural role of 46% of plants in the pollination layer, 35% of pollinators and 33% of seed dispersers. However, plants in the seed dispersal layer did not change their structural role. While most species (73%) changed their role from connector to peripheral, a few shifted from network hub to connector (4%) or module hub (4%) (figure 3).

### (c) Invasive ungulates reduced network stability

**Figure 4.**
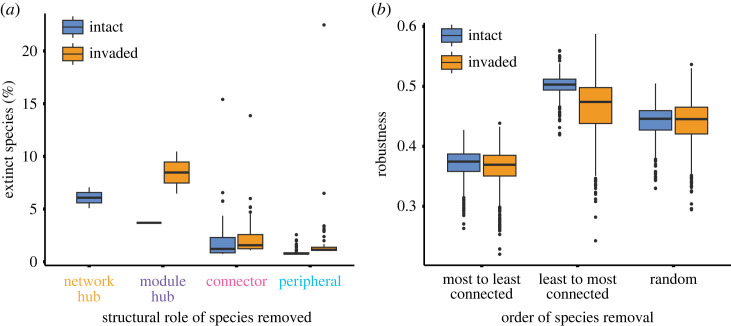
Multitrophic network structure affects stability. Disturbance propagation was higher (*a*), and the robustness was lower (*b*) in the invaded network. (*a*) The disturbance propagation was measured as the proportion of extinct species calculated at equilibrium after removing a single species (*E*, *y*-axis). There were seven (instead of eight) combinations between the structural roles of species removed and network types because there were no network hubs in the invaded network. *E* was affected by the statistical interaction between the network types and the structural role of the removed species. (*b*) Robustness was measured as the area under an extinction curve (AUC). Most-to-least connected removal order: network hub, module hub, connector, peripheral. Least-to-most connected: the opposite order of most-to-least.

The propagation of disturbances through the community was affected by the statistical interaction between the network types and the structural role of the removed species (GLMM, $\chi_{2,231}^{2}=27.28,p<0.01$; figure 4*a*). In the intact network, the removal of network hubs resulted in a 6.9 times higher species extinction than the removal of peripheral species (*post hoc* test, *z* = −8.700, *p* < 0.01). Similarly, removing module hubs produced a 4.7 and 3.2 times higher species extinction than removing peripheral or connector species, respectively, in the invaded network (*post hoc* test, *z* = −4.867, *p* < 0.01 and *z* = 3.182, *p* = 0.03). In addition, on average, the propagation of disturbances produced 1.5 times higher species extinction after removing a single species in the invaded than in the intact network.

Robustness was affected by the interaction between network type and the order of species removal (GLM, $\chi_{2,6000}^{2}=245.9$, *p* < 0.001; figure 4*b*). The invaded network was less robust than the intact network in the least-to-most connected (order: peripheral, connector, module hub, network hub) (*post hoc* test, t ratio=23.16, *p* < 0.001) and most-to-least connected removal orders (*post hoc* test, t ratio=5.16, *p* < 0.001), but not when removing species at random (*post hoc* test, t ratio=0.67, *p* = 0.984).

## 4. Discussion

We studied how invasive species affect the link between structure and stability in a multitrophic mutualistic community. Our empirical approach allowed us to control for the invasion scenario. A previous study found that logging disturbance produced correlated effects between seed dispersal and pollination. However, the indirect links between these two mutualisms and the subsequent effects on stability were not studied [22]. The analytical multilayer approach and dedicated algorithms we applied allowed us to explicitly quantify the propagation of disturbances from one interaction type to another.

Only a few studies have investigated structure and stability in multitrophic communities, and our study contributes to this knowledge in two ways. First, previous studies focused on disentangling how each trophic interaction’s topology separately affects the whole community’s stability [6,8,52]. For example, the stabilizing mechanisms in antagonistic (modularity) and mutualistic (nestedness) networks have a weak effect when considering both interactions [6]. By contrast, we consider the two layers simultaneously. Second, most studies evaluated mutualistic and antagonist interactions (mainly pollinator–plant and plant–herbivory) using simulated data [6,8,52]. One study that used empirical data showed that the combination of properties enhancing robustness in antagonistic (modularity) and mutualistic (nestedness) networks led to an increase in the robustness of a parrot community when including both types of ecological interactions [7]. By contrast, in our networks, both layers were mutualistic interactions obtained from empirical data.

The network had a marked signature of structural reorganization following the invasion. Non-native ungulates reduced the connectivity between pollination and seed dispersal mutualisms and caused link turnover. Link turnover could result from species turnover and/or rewiring of interactions between the same pool of animals and plants in the community [53]. These are two well-documented mechanisms to cope with fluctuation in resource availability in animals [54,55]. We further observed fewer modules with a skewed size distribution in the invaded network. This could result from the local extinction of species and interactions because smaller networks tend to contain fewer modules [51,56]. Local extinction can happen due to the disruption of the keystone interaction and the subsequent changes to the vegetation [24,35]. At the node level, species changed their structural role, primarily from connectors to peripherals. These shifts fragmented the network by reducing the ‘bridges’ among modules [13]. Previous studies also demonstrated that the structural role of species changes in response to disturbances (e.g. fire and habitat loss) [32,57]. In particular, non-native ungulates may reduce the abundance of palatable plants, such as *Ribes magellanicum* and *A. aurea*, altering the role they play in the network structure. Altogether, these results highlight the importance of disturbances on community structure.

Changes to network structure altered the dynamics of cascading effects, increasing the propagation of disturbances and reducing the robustness of the invaded network. In both networks removing connectors had a lower effect than removing module hubs. This result was unexpected because connectors are often seen as important to structural cohesion of networks [58,59]. Nevertheless, disturbance propagation was greater in the presence of non-native ungulates when removing module hubs. A plausible explanation is the presence of a few large and isolated modules containing many peripheral species. Such structure concentrates disturbance propagation within modules and likely produces a high percentage of species extinction after removing a module hub [60,61], collapsing the whole module. Conversely, in the intact network, the large number of connected modules containing a low proportion of species allows disturbance to propagate across the network, limiting the collapse of entire modules. By controlling for network size, we further found that the high number of species in the intact network reduced the propagation of disturbances. A negative correlation between network size and disturbance propagation has been found in mutualistic networks [62].

As with propagation, the intact network was more robust to sequential species removal. This result is consistent with literature showing that a higher number of modules slows down cascading effects through the network, increasing the tolerance of the network to disturbances [15,63]. In particular, the strong robustness to the sequential removal of peripheral species in the intact network indicates a higher community tolerance to common disturbance scenarios such as habitat fragmentation and climate change, primarily affecting rare and specialist species [64].

Our study has two main limitations. First, we uncovered the impacts of invasive species on the structure and dynamics of communities 100 years after the introduction of invasive species. Nevertheless, invasion dynamics encompass different stages (e.g. establishment and spread) in which exotic species interact differently with species in the new community before becoming invasive [65,66]. Hence, we encourage future studies to focus on how exotic species reshape structure and stability across different invasion stages using temporal networks. Second, the stochastic extinction model does not incorporate all ecological processes occurring in natural systems [67,68]. For example, interaction rewiring was not included. Nevertheless, we increased the biological realism by considering the extent of species dependence on the mutualistic interactions using data on species diet [47]. A relevant open question is how different trophic groups respond to extinction cascades. Our preliminary analysis in this direction showed that some trophic groups are more prone to extinction (see ‘Disturbance propagation according to trophic groups’ in the electronic supplementary material for more details).

## 5. Conclusion

Understanding how disturbances reshape the structure of communities and alter their ability to cope with perturbations is essential due to the growing rates of species invasion and biodiversity loss [9,69]. We demonstrated that the disruption of a keystone interaction by invasive species triggers changes to the structure of a multilayer mutualistic network, affecting multitrophic extinction cascades. We encourage future studies to use multilayer networks to understand the response of multitrophic communities to perturbations. While the loss of the keystone interaction erodes the community structure, it underscores the importance of keystone interactions to restore communities. Management practices such as rewilding consider reintroducing extinct keystone species to their historical distribution to restore ecological functions [70]. Our findings highlight that such approaches will benefit from incorporating keystone interactions and recognizing the multitrophic nature of ecological communities.

## Data Availability

Raw data supporting the results are available in the Figshare repository: https://doi.org/10.6084/m9.figshare.15032136 [71]. The code used to process the data and to perform the analyses and simulations is available on Github: https://github.com/Ecological-Complexity-Lab/Poll_Seed-disp_Multilayer_Patagonia.git. The data are also provided in electronic supplementary material [72].
